# Trisomy 1q41-qter and monosomy 3p26.3-pter in a family with a translocation (1;3): further delineation of the syndromes

**DOI:** 10.1186/1755-8794-7-55

**Published:** 2014-09-15

**Authors:** Alicia Cervantes, Constanza García-Delgado, Fernando Fernández-Ramírez, Carolina Galaz-Montoya, Ariadna Berenice Morales-Jiménez, Karem Nieto-Martínez, Laura Gómez-Laguna, Judith Villa-Morales, Mónica Quintana-Palma, Jaime Berúmen, Susana Kofman, Verónica F Morán-Barroso

**Affiliations:** 1 Servicio de Genética, Hospital General de México Dr. Eduardo Liceaga. Facultad de Medicina, UNAM, México, D.F., México; 2 Departamento de Genética, Hospital Infantil de México Federico Gómez, Calle Dr. Márquez 162. Col. Doctores, Del. Cuauhtémoc, 06720 México, D.F., México; 3 Servicio de Genética, Hospital General de México Dr. Eduardo Liceaga, México, D.F., México; 4 Facultad de Medicina, UNAM, México, D.F., México; 5 Departamento de Medicina Genómica, Hospital General de México Dr. Eduardo Liceaga, México, D.F., México; 6 Present address: Departamento de Genética, Instituto Nacional de Perinatología, México, D.F., México

**Keywords:** Chromosomal rearrangement, Trisomy 1q41-qter, Monosomy 3p26.3-pter, Balanced translocation

## Abstract

**Background:**

Trisomy 1q and monosomy 3p deriving from a t(1;3) is an infrequent event. The clinical characteristics of trisomy 1q41-qter have been described but there is not a delineation of the syndrome. The 3p25.3-pter monosomy syndrome (MIM 613792) characteristics include low birth weight, microcephaly, psychomotor and growth retardation and abnormal facies.

**Case presentation:**

A 2 years 8 months Mexican mestizo male patient was evaluated due to a trisomy 1q and monosomy 3p derived from a familial t(1;3)(q41;q26.3). Four female carriers of the balanced translocation and one relative that may have been similarly affected as the proband were identified. The implicated chromosomal regions were defined by microarray analysis, the patient had a trisomy 1q41-qter of 30.3 Mb in extension comprising about 240 protein coding genes and a monosomy 3p26.3-pter of 1.7 Mb including only the genes *CNTN6* (MIM 607220) and *CHL1* (MIM 607416), which have been implicated in dendrite development. Their contribution to the phenotype, regarding the definition of trisomy 1q41-qter and monosomy 3p26.3-pter syndromes are discussed.

**Conclusion:**

We propose that a trisomy 1q41-qter syndrome should be considered in particular when the following characteristics are present: postnatal growth delay, macrocephaly, wide fontanelle, triangular facies, frontal bossing, thick eye brows, down slanting palpebral fissures, hypertelorism, flat nasal bridge, hypoplasic nostrils, long filtrum, high palate, microretrognathia, ear abnormalities, neural abnormalities (in particular ventricular dilatation), psychomotor developmental delay and mental retardation. Our patient showed most of these clinical characteristics with exception of macrocephaly, possibly due to a compensatory effect by haploinsufficiency of the two genes lost from 3p. The identification of carriers has important implications for genetic counseling as the risk of a new born with either a der(3) or der(1) resulting from an adjacent-1 segregation is of 25% for each of them, as the products of adjacent-2 or 3:1 segregations are not expected to be viable.

## Background

There are 14 patients reported with trisomy 1q and monosomy 3p deriving from a t(1;3) which *per se* is an infrequent event (Table 1) [1-7]. The clinical characteristics of distal trisomy 1q syndrome have been described in several cases but a precise characterization of the syndrome has not been achieved (Table 2) [8-21]. In this regard, only 9 cases with a pure trisomy have been reported [8,11,13,14,16-18,20], some of them correspond to small interstitial duplications [14,16-18]; and in 5 of them, a translocation with the short arm of an acrocentric chromosome is implicated [8,11,13,20]. Other cases are derived from an unbalanced translocation that have a small monosomic segment from another chromosome [9,10,12,15,19,21], additionally the proximal break point varies between 1q41 [9,11,15,17,20,21] and 1q42 [8-12,14,18,19], but only two of them have been studied by genomic methodologies [19,21] (Table 2).

**Table 1:** Clinical characteristics of patients with trisomy 1q and monosomy 3p derived from t(1;3)

**Reference**	**Yunis **[1]	**Cook **[2]			**Schinzel **[3]	**McCarthy **[4]			**Sunaga **[5]		**Kozma **[6]			**Li **[7]	**Our study**
1q trisomyc segment/size	q32→qter	q25→qter			q32→qter	q25→qter			q42.3→qter		q42.3→qter			q42.13→qter 21.6 Mb	q41→qter 30.4 Mb
3p monosomyc segment/size	p25→pter	p23→pter			p25→pter	p23→pter			p26.3→pter		p25→pter			p25.3→pter 10.79 Mb	p26.3→pter 1.7 Mb
Cytogenetic analyses	K	K			K	K			HRK		HRK, FISH			K, aCGH	K, FISH, HDMA
Patient		P1	P 2	P 3		P 1	P 2	P 3	P 1	P 2	P 1	P 2	P 3		
Origin	*de novo*	mat	mat	pat	mat	mat	mat	pat	mat	mat	pat	mat	mat	mat	mat
Gender	F	F	M	F	M	F	M	F	M	M	F	F	M	M	M
Age	1 y	NR	NR	NR	17y 8 m	20 y	19 y	18y	6 y	4 y	3 wks	At B	At B	At B	2y 8 m
Age at death	NR	NR	NR	NR	NR	-	-	-	-	-	3 wks	16 m	9 y	9 m	-
Hirsutism	-	NR	NR	NR	+	-	-	-	-	-	-	-	-	+	+
Brachicephaly	+	NR	NR	NR	-	+	NR	NR	NR	NR	+	-	-	-	+
Wide fontanelles	+	NR	NR	NR	-	NR	NR	NR	NR	NR	+	-	-	+	-
CNSA	NR	NR	NR	NR	+	NR	NR	NR	VD	VD	+	+	-	VD	VD
Abnormal EEC	NR	NR	NR	NR	NR	NR	NR	NR	+	-	+	+	+	+	+
Abnormal face	-	+	+	+	TF	+	+	+	-	-	TF	TF	TF	-	+
TI	+	NR	NR	NR	+	NR	NR	NR	+	+	-	-	-	+	+
Epicantal folding	+	NR	NR	NR	+	NR	NR	NR	+	+	-	-	-	NR	-
Hypertelorism	+	NR	NR	NR	+	NR	NR	NR	+	+	+	+	+	NR	+
OEA	+	-	-	-	+	-	-	+	+	+	+	+	+	+	+
Nostrils	-	NR	NR	NR	AN	NR	NR	NR	AN	-	-	AN	AN	AN	AN
Long filtrum	+	NR	NR	NR	-	NR	+	NR	+	+	+	+	+	-	+
Narrow palate	-	NR	NR	NR	+	NR	NR	NR	+	+	+	NR	NR	-	+
Micrognathia	-	NR	NR	NR	-	NR	NR	NR	+	+	+	+	+	+	+
Dysmorphic ears	+	NR	NR	NR	+	+	+	-	+	+	+	+	+	+	+
CHD	-	+	+	+	-	NR	NR	NR	-	-	+	+	-	+	-
GTA	-	NR	NR	NR	-	-	+	-	-	-	-	-	+	+	-
PD/MR	+	+	+	+	+	+	+	+	+	+	+	+	+	+	+

aCGH: array Comparative Genomic Hybridization; AN: ANterverted; At B: At Birth; CHD: Congenital Heart Disease; CNSA: Central Nervous System Abnormalities; EEC: Electroencephalogram; F: Female, FISH: Fluorescent *in situ* hybridization; GTA: Genitourinary Track Anomalies; HDMA: high density microarray; HRK: High Resolution Karyotype; K: Karyotype with banding techniques; M: Male; NR: Non Reported; OEA: Other Eye Abnormalities; PD/MR: Psychomotor Delay/Mental Retardation; TF: Triangular Face TI: Temporal Indentation; VD: Ventricular Dilatation; wks: weeks; y: years, m: months; +: feature present; -: feature absent.

**Table 2:** Clinical characteristics of patients with distal trisomy 1q

**Reference**	**Chia **[8]	**Rasmussen **[9]				**Kennerknecht **[10]	**Verschuuren-Bemelmans **[11]	**Concolino **[12]	**Villa **[13]	**De Brasi **[14]	**Emberger **[15]	**Morava **[16]	**Polityko **[17]	**Cocce **[18]	**Percesepe **[19]	**Kulikowski **[20]	**Shin **[21]	**Our study**
1q trisomic segment	q42-qter^a^	q41- qter^b^	q42-qter^c^	q41-qter^d^	q42-qter^e^	q42-qter^f^	q42.1-qter^g^	q42.1-qter^h^	q44-qter^i^	q42-q44^j^	q41-qter^k^	q43	q41-q44^l^	q42-q43	q42-qter^m^	q41-qter^n^	q41-qter^o^	q41-qter^p^
Monosomic region	22 p12-pter	10 q26-qter	13 q34-qter	4 q34-qter	4 q35-qter	15 qter	15p11.1-pter	8 p23.3-pter	1 p12-pter	none	8 p23.3-pter	none	none	none	9 q34.3-qter	13 p12-pter	11 p15.5-pter	3 p26.3-pter
Cytogenetic analysis	K	K	K	K	K	K	K, FISH	K, FISH	K, FISH, PMM	K, FISH	K, FISH	K, FISH	K, FISH, MCB	K,FISH	K, FISH, aCGH	K, FISH	K, FISH, aCGH	K, FISH, HDMA
Origin	mat	pat	mat	pat	mat	pat	pat	*de novo*	mat	*de novo*	*de novo*	*de novo*	NR	*de novo*	*de novo*	*de novo*	mat	mat
Gender	M	F	F	M	M	M	F/M	F	F	F	F	M	F	F	M	M	M	M
Age	At birth	1 m	21 m	2y	7y 5 m	5y 1 m	22y/21y	6 m	5y	2y 3 m	11y	5y	1y 8 m	4y 9 m	1y	11y	6 m	2y 8 m
PGD	NA	-	+	+	-	+	2/2	-	-	-	+	+	+	+	NR	-	+	+
Macrocephaly	+	+	-^q^	+	+	-^q^	2/2	+	+	+	+	+	-^q^	+	+	+	+	-
Wide fontanelles	+	+	-	+	+	+	+/-	+	-	+	+	NR	NR	NR	+	NR	-	-
CNSA	-	VD, CA	VD,CA	VD	-	-	-/WCM	NR	-	VD	NR	NR	VD	NR	VD	-	NR	VD,SAC
Triangular face	-	-	+	+	-	+	2/2	+	-	+	-	+	NR	+	+	+	+	+
Frontal bossing	+	+	+	+	+	+	2/2	+	+	+	+	+	NR	+	+	+	+	+
T, DS eye brows	+	-	-	+	-	-	2/2	-	-	-	-	NR	+	-	+	-	-	+
DSPF	-	+	+	-	+	-	2/2	+	-	+	+	NR	+	-	+	+	-	-
Ptosis	-	-	+	-	+	-	0/2	-	-	-	-	NR	-	-	+	-	NR	+
Hypertelorism	-	-	+	-	+	-	0/2	-	+	-	-	+	+	+	NR	+	NR	+
OEA	NR	+	-	-	+	-	0/2	NR	-	+	-	NR	-	-	+	-	NR	+
Broad nasal bridge	+	-	-	+	-	-	0/2	-	-	+	+	NR	-	-	+	-	+	+
Hypoplasic nostrils	-	-	-	+	+	-	0/2	-	-	-	-	NR	+	-	-	+	-	-
Long Filtrum	+	+	-	+	+	-	0/2	+	-	-	-	+	-	+	+	-	-	+
High palate	NR	+	+	+	BU	NR	+/-	NR	-	+	-	NR	+	NR	NR	+	NR	+
Micro/retrognathia	+	+	+	+	NR	+	2/2	+	-	+	+	NR	NR	+	+	+	+	+
Dysmorphic ears	+	+	-	+	+	NR	0/2	-	-	-	-	NR	+	+	+	+	+	+
CHD	+	-	-	+	-	-	+/-	-	+	+	-	+	+	-	+	-	-	-
GTA	-	NR	NR	+	-	-	-/+	-	+	-	-	NR	NR	-	+	-	-	-
Limb abnormalities	-	-	+	+	+	+	+/-	-	-	+	+	NR	+	+	+	+	NR	+
CH	+	+	-	+	-	-	0/2	+	-	-	-	NR	NR	NR	NR	NR	-	-
PD/MR	NA	+	+	+	+	+	2/2	+	-	+	+	+	+	+	+	+	+	+
Abnormal Language	NA	+	NR	+	+	NR	NR	NA	NR	+	-	NR	+	NR	NR	+	NR	+

^a^: 46,XY,der(22)t(1;22)(q42;p12); ^b^: 46,XX,der(10)t(1;10)(q41;q26); ^c^: 46,XX,der(13)t(1;13)(q42;q34); ^d^: 46,XY,der(4)t(1;4)(q41;q34); ^e^: 46,XY,der(4)t(1;4)(q42;q35); ^f^: 46,XY,der(15)t(1;15)(q42;qter); ^g^: 46,XX,der(15)t(1;15)(q42,1;p11.1); ^h^: 46,XX,der(8)t(1;8)(q42.2;p23.3) at amniocentesis; ^i^: 46,XX,der(15)t(1;15)(q44;p12); ^j^: 46,XX,dup(1)(q44q42), ^k^: 46,XX,der(8)t(1;8)(q41;p23.3) ^l^: 46,XX,inv dup(1)(q41-q44); ^m^: 46,XY,der(9)t(1;9)(qter-q41::q34.3-pter); ^n^: 46,XY,der(13)t(1;13)(q41;p12) ^o^: 46,XY,der(11)t(1;11)(q41;p15.5)mat; ^p^: 46,XY,der(3)t(1;3)(q41;p26.3); ^q^: microcephaly; aCGH: Array Comparative Genomic Hybridization; BU: Bifid Uvula; CA: Cortical Atrophy; CH: Capillary Hemangiomas; CHD: Congenital Heart Disease; CNSA: Central Nervous System Abnormalities; DSPF: Down Slanting Palpebral Fissures; F: Female; FISH: Fluorescent *in situ* hybridization; GTA: Genitourinary Track Abnormalities; HDMA: high density microarray; K: Karyotype with banding techniques; M: Male, m: months; mat: maternal; MCB: MultiColor Banding; PD/MR: Psychomotor Delay/Mental Retardation; NA: Non Applicable; NR: Non Reported; OEA: Other Eye Abnormalities; pat: paternal; PMM: Polymorphic Microsatellite Markers; PGD: Postnatal Growth Delay; T/DS: Thick/Down Slanting; VD: Ventricular Dilatation; y: years; WCM: Wide Cisterna Magna; +: feature present; -: feature absent.

Several cases of 3p25.3-pter monosomy syndrome (MIM 613792) have been delineated (Table 3) [22-29]. The clinical manifestations of monosomy 3p syndrome include low birth weight, microcephaly, trigonocephaly, hypotonia, psychomotor and growth retardation, among others (Table 3). Although intellectual deficits are almost invariably associated with cytogenetically visible 3p deletions, patients with infrequent 3p25-p26 or terminal deletions display normal intelligence or mild abnormalities [26,27,30]. A critical region has been identified for monosomy 3p and several genes have been proposed as responsible for the phenotypic features [24,25,30], however none critical region or candidate genes have been identified for terminal trisomy 1q syndrome [12,20,21,31]. We report the case of a patient with a trisomy 1q and monosomy 3p derived from a familial t(1;3)(q41;q26.3). The chromosomal regions involved were defined by high-density microarray techniques and their effects in the phenotype regarding the definition of the syndromes are discussed.

**Table 3:** Clinical characteristics of patients with monosomy 3p with different break points and sizes

**Reference**	**Fernandez **[22]	**Fernandez **[23]	**Gunnarson **[24]	**Shuib **[25]				**Pohjola **[26]		**Cuoco **[27]	**Chen **[28]	**Peltekova **[29]	**Our study**
3p monosomic segment/ Size	p26^a^ none	p26.2-pter^b^ 4.5 Mb	p25.3-p26.1^c^ 1.6 Mb	p25.2-pter 12.65 Mb, 12.25 Mb, 12.05 Mb	p25.3-pter 9.55 Mb to 11.50 Mb	p25.3-p26.1 6.3 Mb	p26.1-pter 8.6 Mb	p25.3-pter 9.0 Mb	p26.3-pter 1.10 Mb	p26.3-pter 0.90 Mb	p25.3-pter 9.3 Mb	p25.3-p25.3 0.643 Mb	p26.3-pter 1.70 Mb
Cytogenetic analysis	K, FISH, SNPa	K, FISH, aCGH	K, SNPa	K, MLPA, SNPa				K, FISH, SNPa, MLPA, SEQ		K, FISH, SNPa,MLPA	K, aCGH, QF-PCR^d^	K, FISH, aCGH, Q-PCR	K, FISH HDMA
Number of patients				3	9					2			
Origin	*de novo*	*de novo*	*de novo*	*de novo*	*de novo*	*de novo*	*de novo*	mat^e^	mat^f^	pat^g^	*de novo*	NR	mat
Gender	M	M	F	NR	NR	NR	NR	M	M	M/M	F	F	M
Age	7y 11 m	5y 6 m	4y	NR	NR	NR	NR	12y	12y	9y/7y	24wg	22y †	2y 8 m
PGD	+	+	-	NR	NR	NR	NR	-	+	0/2	NA	+	+
Hypotonia	+	-	+	NR	NR	NR	NR	+	-	0/2	NA	-	+
Hirsutism	+	-	-	NR	NR	NR	NR	-	-	0/2	NR	-	+
Microcephaly	-	+	+	NR	2/9	+	NR	-	+	0/2	NR	+	-
CNSA	-	-	+	NR	NR	NR	NR	-	-	1/2	-	+	+
Triangular face	-	+	-	NR	NR	NR	NR	-^g^	-	0/2	NR	+	+
DSPF	+	+	-	NR	NR	NR	NR	-	-	0/2	NR	+	-
Ptosis	+	-	+	NR	2/9	+	NR	-	-	0/2	NR	+	+
Hypertelorism	+	+	+	NR	NR	NR	NR	-	-	0/2	+	-	+
OEA	+	NR	+	NR	NR	NR	NR	-	-	1/2	NR	+	+
Broad nasal bridge	+	+	+	NR	NR	NR	NR	+	-	0/2	NR	+	+
Hypoplasic nostrils	-	+	-	NR	NR	NR	NR	-	-	0/2	NR	-	-
High or cleft palate	-	+	-	NR	NR	NR	NR	+	-	0/2	NR	+	+
Micrognathia	-	+	-	NR	2/9	NR	NR	-	-	0/2	+	-	+
Dysmorphic ears	+	+	+	NR	NR	NR	NR	+	-	0/2	+	+	+
CHD	-	-	+	3/3	2/9	-	-	-	-	0/2	-	+	-
GTA	-	+	-	NR	NR	NR	NR	-	-	0/2	NR	-	-
Limb abnormalities	+	+	+	NR	NR	NR	NR	+	+	0/2	NR	+	+
PD/MR	BL	+	+	3/3	9/9	+	-	LD	LD	BL/-	NA	+	+
Abnormal Language	+	+	+	NR	NR	NR	NR	+	+	2/2	NA	+	+
Seizures	-	+	+	NR	NR	NR	-	-	-	1/2	NA	+	+

^a^: 46,XY,t(3;10)(p26;q26), disruption of *CNTN4*; ^b^: 45,XY,der(3)t(3;13)(p26.2;q12.13),-13; ^c^: *In vitro* fertilization; ^d^: amniocentesis and pregnancy interruption; ^e^: mother and sister with the same deletion; ^f^: mother with the same deletion without clinical data and mildly dysmorphic features; ^g^: father with same deletion and no clinical data; ^h^: asymmetric face; aCGH: array Comparative Genomic Hybridization; BL: BorderLine; DSPF: Down Slating Palpebral Fissures; F: Female; FISH: Fluorescent *in situ* hybridization; GTA: Genitourinary Track Anomalies; HDMA: high density microarray; K: Karyotype with banding techniques; LD: Learning Disability; M: Male; m: months; NA: Not Applicable; NR: No Reported; SEQ: sequencing of candidate genes; PD/MR: Psychomotor Delay/Mental Retardation; PGD: Postnatal Growth Delay; QF-PCR: Quantitative Fluorescent-PCR; SNPa: Single Nucleotide Polymorphism array; wg: weeks of gestation; y: years; †: dead at the time of report; +: feature present; -: feature absent.

## Case presentation

### Clinical report

**Figure 1:**
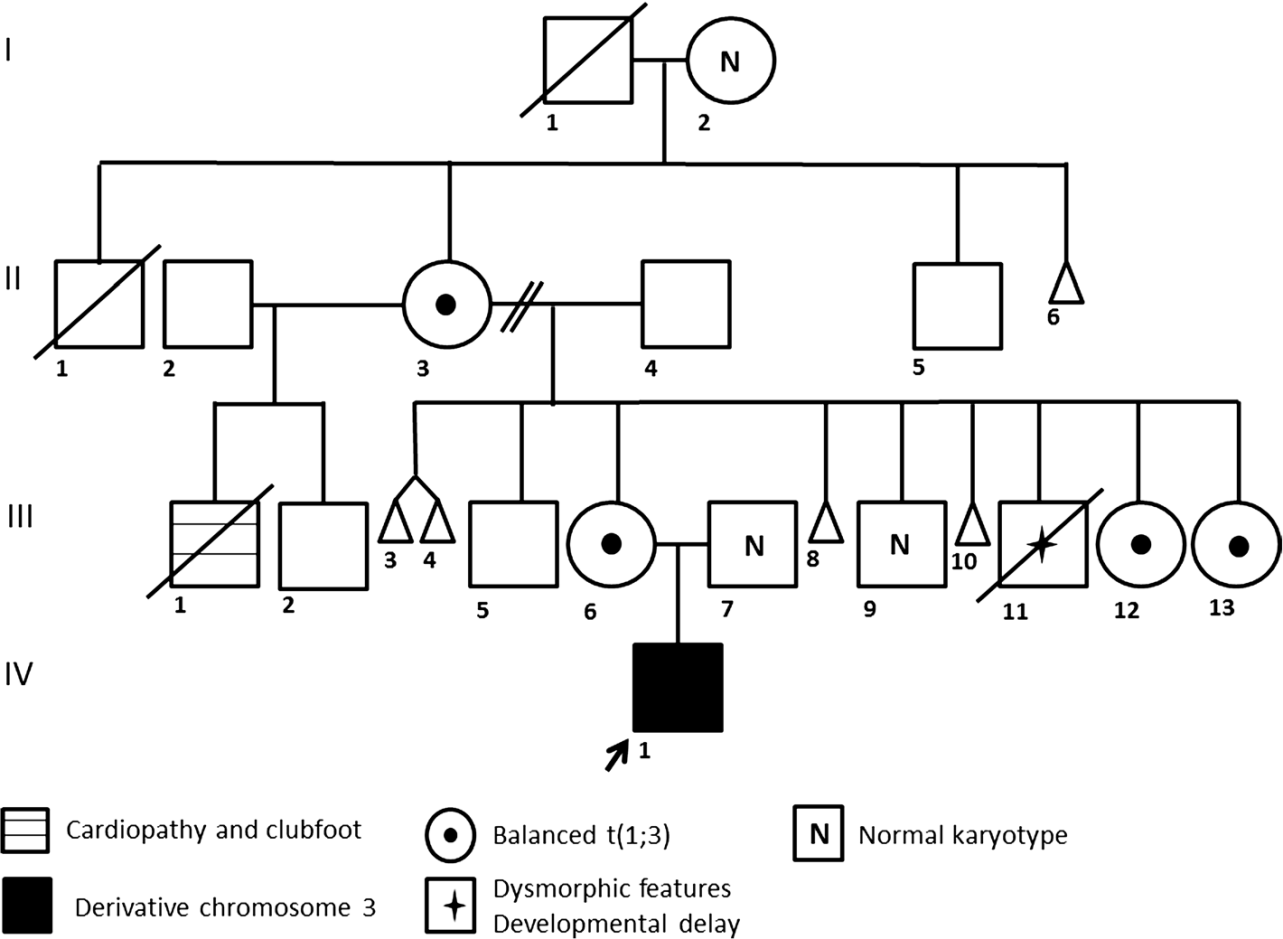
Pedigree of the family.

**Figure 2:**
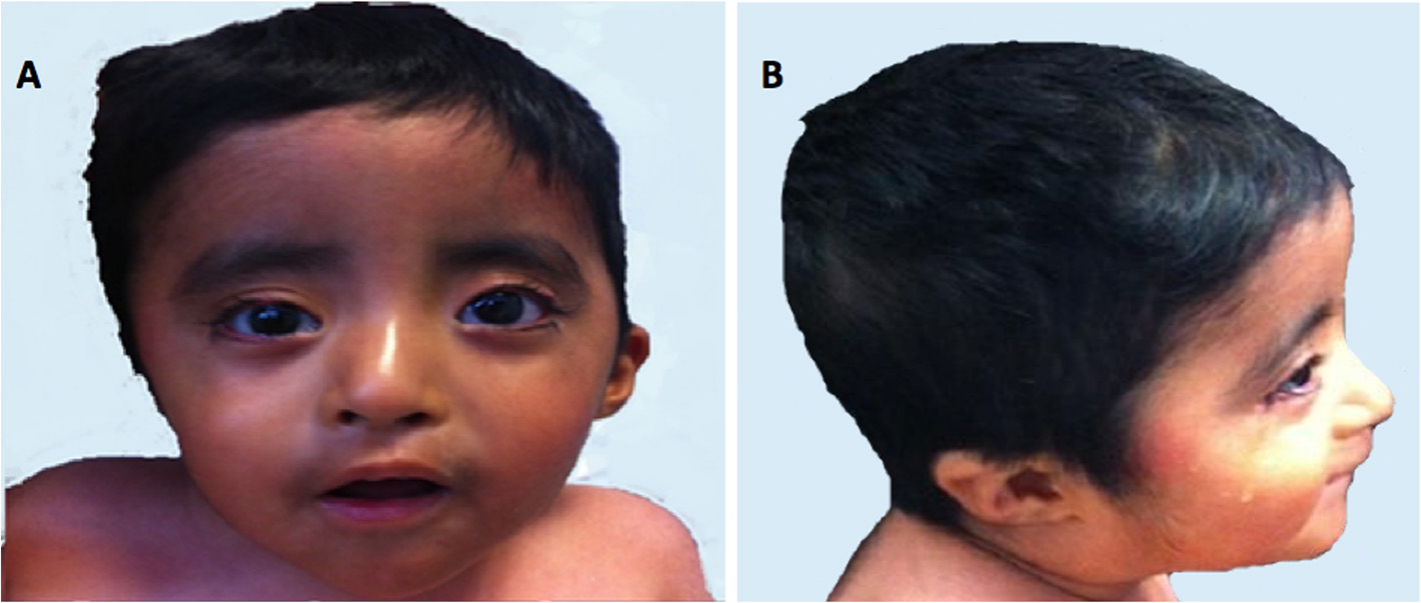
**Facial characteristics of the proband are shown. A)** Frontal view: triangular face and horizontal palpebral fissures. **B)** Lateral view: micrognathia, and posteriorly rotated low set ears.

The proband is a 2 years 8 months Mexican mestizo male (Figure 1, IV.1), first known at 4 months of age due to dysmorphic features and mental development arrest. He is the only child of a young, apparently healthy and unrelated couple. He had two maternal uncles who died during childhood and presented congenital diseases, one of them also had dysmorphic features. The pregnancy was 38 weeks long and was complicated by a threat of miscarriage in the first trimester. His weight was 2,880gr (P10), height 51 cm (P50), and Apgar score 8/9. He could sit without support at 12 months of age; however so far he has not achieved speech, cannot walk and does not control sphincters. He suffered from esophageal reflux at 5 months of age, pneumonia at 8 months and was treated for dacryostenosis at 12 months. At present he has a weight of 11 kg (<P5), height 84 cm (<P5) and head circumference of 48 cm (P25). He has brachicephaly, triangular facies, horizontal palpebral fissures, micrognathia and several dysmorphic features, low set and retroposition of the ears, widely spaced nipples and hypotrophic limbs (Figure 2 and Tables 1, 2 and 3). Heart and renal malformations were discarded. The MRI showed ventriculomegaly and a subarachnoid cyst.

### Cytogenetic analysis

**Figure 3:**
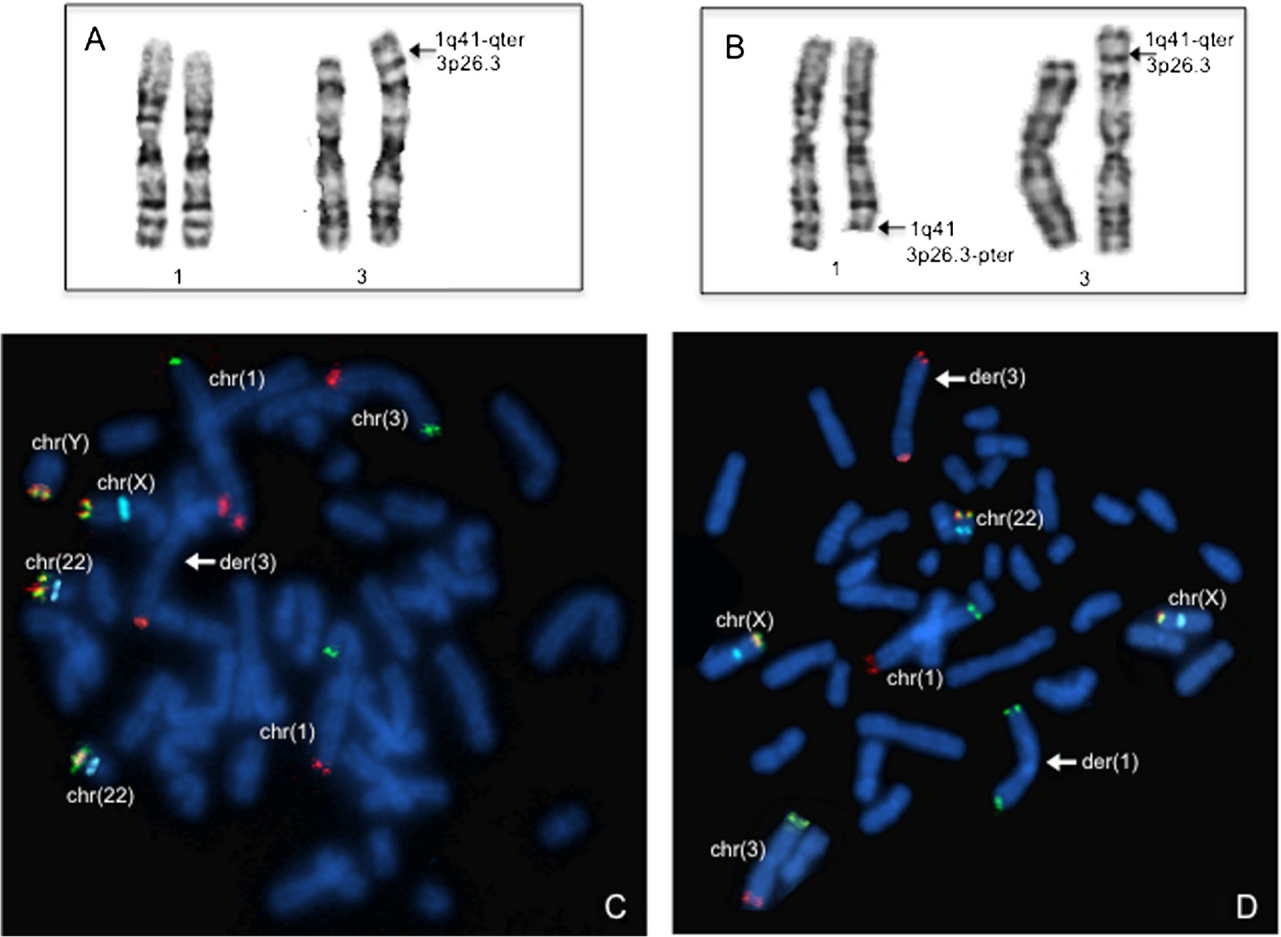
**Cytogenetic analysis. A**. Partial karyotype of the proband showing additional material in 3p26.3. **B**. Partial karyotype of mother’s patient (individual III.6) showing a balanced t(1;3)(q41;p26.3). **C**. FISH analysis in patient’s metaphase showing a der(3) with both subtelomeric probes 1q and 3q in orange (the arrow indicates the der(3) and normal chromosomes 1 and 3 are indicated). **D**. FISH analysis in a metaphase of individual III.6 showing a balanced translocation, the der(1) with subtelomeric 1p and 3p probes both in green and the der(3) with 1q and 3q probes in orange are indicated with arrows, the normal chromosomes 1 and 3 are also indicated.

The GTG banding demonstrated additional material on 3p26.3 in the proband (Figure 3A), the karyotype of the mother (Figure 1, III.6) revealed a balanced translocation between chromosomes 1 and 3 (Figure 3B), also present in other three female family members (Figure 1) with a chromosomal complement 46,XX,t(1;3)(q41;p26.3). FISH analyses using subtelomeric probes (ToTelVysion™, Vysis Abbott, Inc. Abbott Park, IL, USA), mixture 1:1p (CEB108/T7, green), 1q (D1S3738, orange) and mixture 3: 3p (D3S4559, green), 3q (D3S4560, orange) confirmed the presence of the derivative chromosome 3 in the proband and showed the balanced translocation in his mother. Additionaly, mixture 1 includes Xp/Yp (yellow) and centromeric X (aqua) probes, while mixture 3 contains probes 22q (yellow) and *BCR* (aqua) (Figure 3C and D). The final chromosomal formula for the patient was 46,XY,der(3)t(1;3)(q41;p26.3)mat.ish der(3)t(1;3)(D1S3738+,D3S4559-,D3S4560+) and 46,XX,t(1;3)(q41;p26.3)mat.ish t(1;3)(CEB108/T7+,D1S3738-,D3S4559+;D1S3738+, D3S4559-,D3S4560+) for his mother.

### Microarray analysis

**Figure 4:**
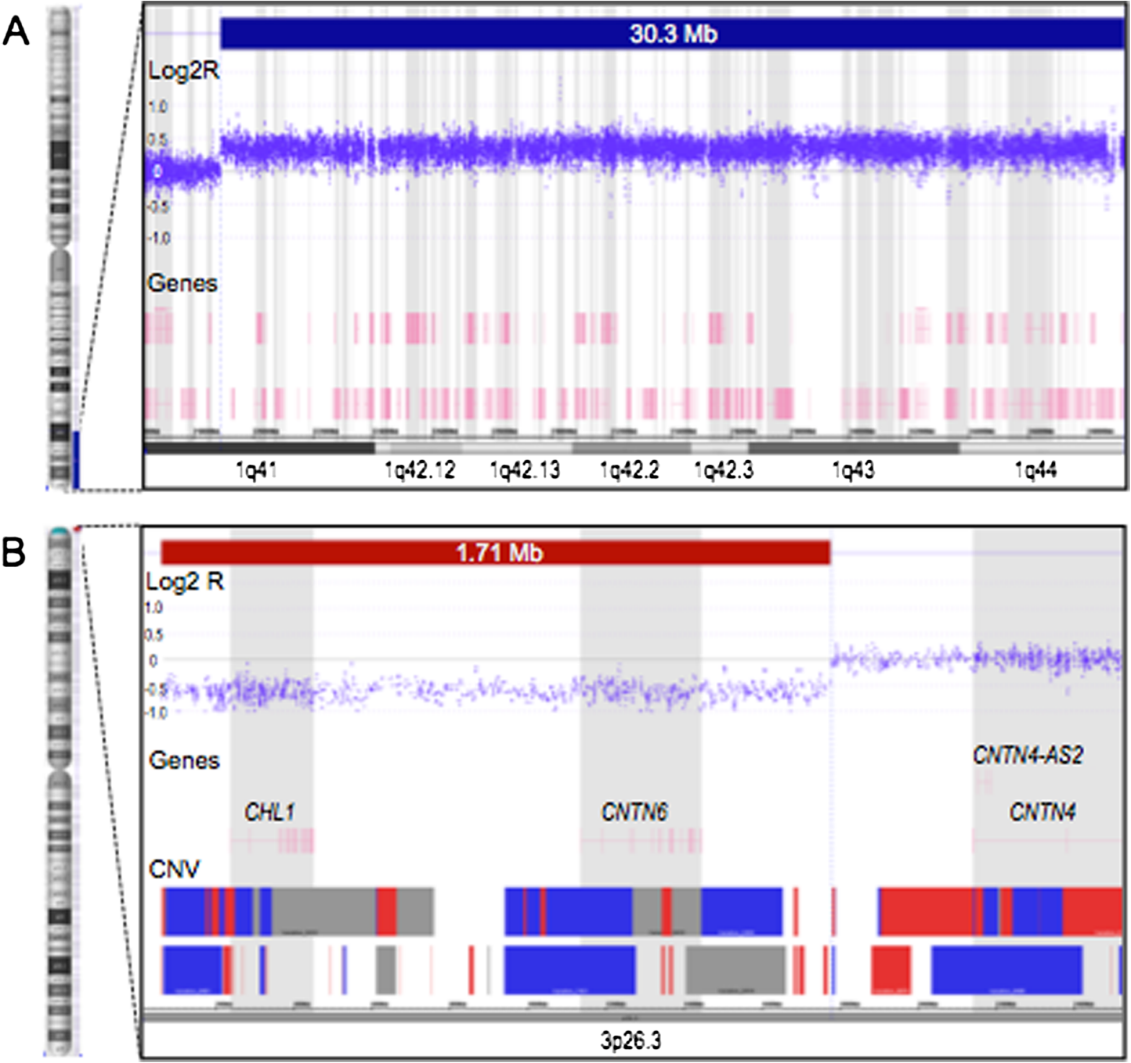
**Microarray analysis. A**. The 30.3 Mb duplication in 1q41-qter and **B**. The 1.71 Mb deletion in 3p26.3-pter identified on the proband by microarray mapping. The solid bars at the top of each panel indicate the copy-number altered regions and chromosome bands are annotated at the bottom. The chromosome scheme at the left of each panel is not to scale.

Genomic DNA was extracted from a peripheral blood sample. Copy-number analysis was performed using the Cytoscan HD array kit (Affymetrix Inc., Santa Clara, CA, USA). Data was analyzed with Chromosome Analysis Suite 2.0 (Affymetrix Inc.), using 50 markers and 200 kb resolution. Joining algorithm was applied for copy-number altered segments interrupted by <200 kb normal state data. Mapping was based on the human genome assembly Feb 2009 (GRCh 37/hg19). The analysis on the proband revealed a chromosome 1q41-qter duplication (pos.218,920,024-249,224,684) and a chromosome 3pter-p26.3 deletion (pos.61,891-1,774,215) (Figure 4). In addition, a 640 kb duplication was detected on chromosome 14q32.33 (pos.106,072,250-106,712,665), this alteration corresponds to a highly represented CNV region, according to the Database of Genomic Variants [32,33].

## Discussion

The patient described in this report has a trisomy 1q41-qter of 30.3 Mb in extension (comprising about 240 protein coding genes) and has a monosomy 3p26.3-pter of only 1.7 Mb. These break points are similar to those found in at least 6 reported cases with der(3)t(1;3) (Table 1) [5-7]. It is interesting to note that 14 patients have been described with trisomy 1q and monosomy 3p, but only one of these cases has been studied by genomic techniques (aCGH) [7]. The case reported here has been addressed by high-density microarray analysis, and revealed different break points and chromosomal segment sizes than the previously published. We consider that our patient shows a phenotype that resembles trisomy 1q41-qter syndrome, as he has brachicephaly, triangular face, temporal indentation, hypertelorism, anteverted nostrils, low set and malformed ears, micrognathia and narrow palate, all of which are features of distal trisomy 1q syndrome [34]. Despite this, we consider that the small deleted region at chromosome 3p could be modifying the phenotype of our patient. For example, head circumference data reported for trisomy 1q shows that most of the patients (82%) have macrocephaly (Table 2) while pure monosomy 3p patients have microcephaly more frequently (53-70%) (Table 3). In this regard, our patient displayed normal head circumference, and considering the specific chromosomal regions implicated in this phenotype and therefore the genes within these *loci* (Table 2), it seems that macrocephaly is associated to a duplicated region close to or in 1q43 [13,14,16-19]. Recently, 1q43 has been proposed as a candidate region for microcephaly, in particular *AKT3* has been considered as a candidate gene for this feature in patients with a microdeletion of 1q43-q44 [35]. On the other hand, according to the described patients with monosomy 3p, both proximal and distal deletions to 3p26 show microcephaly and so far a critical region has not been identified [24-26,29]. Although this characteristic in neither of the two pure syndromes is always present, it is possible that a compensatory effect of both implicated regions could be taking place in our patient and, as observed in other patients with a der(3) of a t(1;3) (Table 1), he has not macro or microcephaly but he has brachicephaly. However, these malformations may be multifactorial in their etiology.

The specific effect of each of the chromosomal regions regarding a particular clinical characteristic cannot be extended to all the features in the patient reported here or in the previously described patients, as this analysis is further complicated by: 1) the precise *loci* involved, 2) the size of the chromosomal fragments implicated in both the translocations and the pure trisomy 1q and monosomy 3p, and 3) the number and function of the genes present. As a result, most of the alterations of the diverse cases are privative of a given patient. When considering the first eight cases reported with a der(3)t(1;3) (Table 1) [1-4], 50% have break points with a longer chromosome fragment implicated than in our patient. In fact, the patients with a more similar phenotype to our patient are the ones reported by Sunaga et al., [5] which have break points in 1q42 and 3p26. However, not even in these cases the similarity is complete as these patients show the brachicephaly and abnormalities of the neural central system (that similar to our case, one of them is ventricular dilatation). All these patients show a triangular face and temporal indentation, but they do not show hirsutism, while some other characteristics are not constant among the three cases (Table 1) [5]. Another interesting consideration is that these patients do not have congenital heart disease and all of them have mental retardation. A critical region for congenital heart disease has been designated to a 0.65 Mb region in 3p25 and four candidate genes have been proposed: *SLC6A1* (MIM 137165)*, HRH1* (MIM 600167)*, ATG7* (MIM 608760) and *CRELD1* (MIM 607170) [24,25,30]. This region is not implicated in our case (Tables 1 and 3) and this may explain why even though both chromosomal abnormalities involve heart disease, our case does not show this characteristic. Only a third of the patients with terminal trisomy 1q have congenital heart disease and apparently, there is not a critical region for this malformation (Table 2) [8,9,11,13,16,17].

Microarray analysis on our patient demonstrated that two genes, *CNTN6* (MIM 607220) and *CHL1* (MIM 607416) (which has been previously implicated in neuronal development associated to dendrite migration and mental retardation), are located within the deleted region and might implicate an haploinsufficiency mechanism, as loss of these genes has been related to mild mental deficit or do not present symptoms at all [26,27]. Studies about *CNTN4* (MIM 607280) indicate that it belongs to the same family as *CNTN6.* In a patient described by Fernandez et al., [22], *CNTN4* was disrupted by a balanced translocation, while the patient displayed severe clinical manifestations associated to monosomy 3p (in particular the facial features without triangular face or microcephaly) and mental retardation. Assuming that even if no chromosomal material was gain or lost in the case referred, *CNTN4* is an important gene for brain development. Therefore, it has been considered that *CNTN4* corresponds to the critical region or is one critical gene for the monosomy 3p clinical characteristics. In our patient, *CNTN4* is not included in the deleted region as it is further centromeric, however *CNTN6* is deleted and, considering that it belongs to the same gene family, its effect should not be underestimated. In addition, studies by Mercati et al., [36] have shown that *CNTN6* is implicated in the same pathway as *CHL1*, i.e. the integrity of the neuronal development. *CHL1* gene participates in brain development, interacting with a tyrosin phosphatase protein, regulating its activity in the complex signaling of the neuronal dendritic migration. Therefore, its deletion may have an important role in the mental retardation observed in our patient, and although its importance is difficult to assess, as in the patients described by Pohjola et al., [26] and Couco et al., [27] in whom these genes are lost, some of them have a borderline development, learning disabilities or are apparently normal. It is possible that in our patient, due to the presence of the trisomy 1q41-qter, the haploinsufficiency of both genes could have a synergic effect that in turn compromises the psychomotor development.

Analysis of the phenotypic impact that the chromosome 1q trisomy region may have on our patient is complicated by the fact that over 240 genes may be involved, and in general terms the additive effect of these genes may be related to the facial, cranial and cognitive abnormalities (Table 2). Although several cases with similar chromosomal abnormalities have been described, the contribution of specific regions in the development of the characteristics of the syndrome is not clear, due to: 1) the high number of genes implicated, 2) the variability of the break points and 3) the fact that not all of the cases correspond to pure terminal trisomies. Therefore, a trisomy 1q41-qter syndrome should be considered in particular when the following characteristics are present: postnatal growth delay, macrocephaly, wide fontanelle, triangular face, frontal bossing, thick eye brows, down slanting palpebral fissures, hypertelorism, flat nasal bridge, hypoplasic nostrils, long filtrum, high palate, microretrognathia, ear abnormalities, neural abnormalities (in particular ventricular dilatation), psychomotor delay and mental retardation (Table 2).

In the 1q41-q42 region are the *loci* for 5S rRNA and this situation may predispose to chromosomal rearrangements, in particular with the short arms of acrocentric chromosomes during the transcription of ribosomal genes [37]. This is illustrated in Table 1, as in 5 out of 9 cases with pure 1q trisomy, this region is implicated [8,11,13,20]. Furthermore, recently a new case of trisomy 1q41-qter has been described in a patient who also has a partial trisomy 9pter-9q21.32, derived from a 3:1 segregation of a maternal balanced translocation, and it has been suggested that the break-point is associated with the presence of a fragile site (FRA1H) localized to 1q41-q42.1 [38].The chromosomal analysis of our family revealed 4 female carriers of the balanced translocation (Figure 1) and this has an important implication for genetic counseling. The pedigree shows 5 cases of spontaneous abortions, which might have resulted from an adjacent-1 segregation. There is also an uncle of the proband (Figure 1, III.11) that was described with psychomotor delay and dysmorphic features, and who died in infancy of unknown causes. We may suppose that this person had a der(3)t(1;3) resulting of adjacent-1 segregation, as with the proband. When considering the segregation of the chromosomes implicated in the translocation, the risk of a newborn with either a der(3) or der(1) resulting from an adjacent-1 segregation is of 25% for each of them, as the products of adjacent-2 or 3:1 segregations are not expected to be viable.

## Conclusion

The case described here gives new insights into the complex definition of distal trisomy 1q and monosomy 3p26.3 syndromes. Even though in this case the trisomy affects such a large quantity of genes, it is possible to evaluate the clinical characteristics in order to understand their participation in the pathogenesis of the syndrome and has very important implications for the genetic assessment.

### Consent

Written informed consent from the patient’s mother for publication of this Case report and any accompanying images. A copy of the written consent is available for review by the Editor of this journal.

## Competing interest

The authors declare that they have no competing interest.

## Author’s contribution

AC carried out the general analysis of the case and drafted the manuscript. CGD, CIGM and VFMB provided the clinical evaluation and VFMB also drafted the manuscript. ABMJ, JVM, MQP carried out the cytogenetics analysis. KNM and LGL carried out the FISH studies. FFR and JB carried out the microarray analysis. SKE participated to the critical reading of the manuscript. All authors read and approved the final manuscript.

## Pre-publication history

The pre-publication history for this paper can be accessed here:

http://www.biomedcentral.com/1755-8794/7/55/prepub
